# Monitoring of cerebrovascular pressure reactivity in children may predict neurologic outcome after hypoxic-ischemic brain injury

**DOI:** 10.1007/s00381-022-05579-4

**Published:** 2022-06-09

**Authors:** Julian Zipfel, Dorothea Hegele, Konstantin Hockel, Susanne R. Kerscher, Ellen Heimberg, Marek Czosnyka, Felix Neunhoeffer, Martin U. Schuhmann

**Affiliations:** 1 Section of Pediatric Neurosurgery, Department of Neurosurgery, University Hospital of Tuebingen, Hoppe-Seyler-Str. 3, 72076 Tuebingen, Germany; 2 Department of Spine Surgery, Isar Klinikum, Sonnenstr. 24-26, Munich, Germany; 3 Pediatric Intensive Care Unit, University Children’s Hospital of Tuebingen, Hoppe-Seyler-Str. 3, 72076 Tuebingen, Germany; 4 Department of Clinical Neurosciences, Division of Neurosurgery, Cambridge University Hospital, Hills Road, Cambridge, UK

**Keywords:** Autoregulation, Cerebrovascular reactivity, Hypoxic-ischemic brain injury, Non-traumatic brain injury

## Abstract

**Objectives:**

Impaired cerebral blood flow is a first-line reason of ischemic-hypoxic brain injury in children. The principal goal of intensive care management is to detect and prevent further cerebral blood flow deficits. This can be achieved by actively managing cerebral perfusion pressure (CPP) using input from cerebrovascular autoregulation (CAR). The main objective of the current study was to investigate CAR after cardiac arrest in children.

**Methods:**

Nineteen consecutive children younger than 18 years after cardiopulmonary resuscitation, in whom intracranial pressure (ICP) was continuously measured, were included. Blood pressure and ICP were continuously monitored via ICM + software and actively managed using the pressure reactivity index (PRx) to achieve and maintain an optimal CPP. Outcome was scored using the extended Glasgow outcome scale (eGOS) at discharge and 6 months.

**Results:**

Eight children died in hospital. At 6 months, further 4 children had an unfavorable (eGOS1–4) and 7 a favorable (eGOS5–8) outcome. Over the entire monitoring period, we found an elevated ICP (24.5 vs 7.4 mmHg), a lower CPP (50.3 vs 66.2 mmHg) and a higher PRx (0.24 vs − 0.01), indicating impaired CAR, in patients with unfavorable outcome. The dose of impaired autoregulation was significantly higher in unfavorable outcome (54.6 vs 29.3%). Analyzing only the first 72 h after cardiac arrest, ICP ≥ 10 mmHg and PRx > 0.2 correlated to unfavorable outcome.

**Conclusions:**

Significant doses of impaired CAR within 72 h after resuscitation are associated with unfavorable outcome. The inability to restore autoregulation despite active attempts to do so as well as an elevated ICP may serve as a bad prognostic sign indicating a severe initial hypoxic-ischemic brain injury.

**Supplementary information:**

The online version contains supplementary material available at 10.1007/s00381-022-05579-4.

## Introduction

Cardiac arrest can lead to primary ischemic-hypoxic brain injury, but also secondary brain injury can ensue due to pronounced ischemia, autoregulatory failure, hypoperfusion, blood–brain barrier breakdown, seizures, oxidative injury and hyperpyrexia [1]. Critically low cerebral blood flow during cardiac arrest and initial resuscitation are the primary reasons of hypoxic-ischemic brain injury [2]. Secondary damage during the first days on ICU, however, is avoidable. To do so, it needs to be detected and prevented [2].

In traumatic brain injury (TBI), time dose spent with impaired autoregulation correlates with neurological outcome [3], and cerebral hypoperfusion is a well-described mechanism leading to secondary brain damage [4].

In TBI, the pressure reactivity index (PRx), which is calculated as the correlation coefficient between intracranial pressure (ICP) and arterial blood pressure as described before [5, 15], has repeatedly been validated as a marker of cerebral autoregulation [5]. This parameter correlates strongly with unfavorable neurological outcome and identifies individual thresholds for the cerebral perfusion pressure (CPP) [6–8] in both adult and pediatric patients [9–12]. We previously showed that independent assessment and management of CAR are feasible in pediatric TBI and that a higher dose of impaired autoregulation is strongly correlated with unfavorable outcome [3]. In adults, scarce data exists on ICP monitoring after hypoxic-ischemic brain injury. A single study did not find pathological ICP, but impaired intracranial compliance [13]. In many centers, ICP monitoring is not part of routine care for patients having sustained cardiac arrest and probable hypoxic-ischemic brain injury. Recently, Balu et al. were able to show that elevated ICP and PRx are associated with unfavorable outcome in adult patients after cardiac arrest [14].

In our institution, we implemented a post-resuscitation care protocol for children who likely sustained low-flow brain injury. This includes the neurosurgical insertion of an intraparenchymal intracranial pressure (ICP) probe. Univariate ICP management alone might not be able to prevent secondary brain damage. Maintenance of this adequate—optimal—CPP might be of importance, as already shown in adult TBI [3, 8, 15]. No CPP guidelines for hypoxic-ischemic brain injury exist.

In a former pilot study in pediatric patients after cardiac arrest, we investigated if similar mechanisms and relations exist in non-traumatic hypoxic-ischemic brain injury following resuscitation [16]. This study suggested that time spent with impaired autoregulation correlates with neurological outcome. Our goal for this follow-up study was to investigate in a larger cohort and in more detail the value of CAR monitoring in the setting of pediatric patients after cardiac arrest with probable significant low flow time and re-analyze the data with more statistical power. The primary objectives were to confirm that results of CAR monitoring via PRx (time of bad autoregulation) correlate with clinical outcome in children after cardiac arrest and to evaluate the effect of our institutional CPP optimization protocol.

## Materials and methods

Extending our initial (2013–2016) cohort of 11 consecutive children after cardiopulmonal resuscitation and clinical signs of significant cerebral low-flow time (e.g. prolonged CPR duration or time without CPR, imaging findings of cerebral swelling, intermittently dilated pupils, intubation and sedation), we included 8 further patients treated between 2017 and 2020 on our pediatric ICU. Adhering to our internal guidelines, all children initially received a cranial computed tomography, were afterwards immediately admitted to our pediatric ICU and a right frontal intraparenchymal pressure transducer was placed into the white matter at a depth of 3 cm. This was performed as soon as core temperature and coagulation parameters were normalized. In 4 of 19 cases, this could not be achieved within a few hours after admission, and a delay of > 24 h occurred until monitoring started.

We performed active management of blood pressure and ICP, and thus CPP, to determine and then maintain the optimal CPP (CPPopt) using PRx for this purpose as described before [3]. All patients were intubated, mechanically ventilated and under deep analgo-sedation. A thorough overview of our therapeutic protocol is provided as supplemental material. Before discharge, a cranial magnetic resonance imaging (MRI) scan was performed to visualize the extent of cerebral ischemia. Outcome assessment was done using the extended Glasgow Outcome Scale (eGOS) at discharge from hospital, 3 months, 6 months and 12 months after the event.

Inclusion criteria were as follows: admission to the pediatric ICU after cardiac arrest, age < 18 years at the time of admission, absence of laboratory or clinical coagulopathy and signs of significant hypoxic-ischemic brain injury like Glasgow Coma Scale < 8, dilated pupils, prolonged resuscitation. Excluded from our analysis were children who did not receive an ICP probe.

Patients were dichotomized according to eGOS at 6 months into a favorable outcome (eGOS 5–8) and an unfavorable outcome (eGOS 1–4) group.

Arterial blood pressure was continuously monitored and referenced to the level of the foramen of Monro. Monitoring parameters were digitally sampled via ICM + software (Cambridge Enterprise, Cambridge, UK). CPP and PRx were calculated as described previously [5, 15]. PRx is calculated via ICM + software as the Pearson correlation coefficient between 30 consecutive 10-s time averages of ICP and MAP over a 5-min period. PRx is calculated every minute and presented as time trend together with ICP, MAP and CPP.

Mean overall ICP, CPP and PRx values were compared to outcome. Neuroradiological MRI reports were assessed for signs of hypoxic-ischemic lesions and dichotomized as physiological and pathological (severe changes: lesions with high DWI signal, hyperintensity on T2 and FLAIR especially in the basal ganglia and cortex, sometimes with relative sparing of the perirolandic cortex and thalami; moderate changes: DWI image showing high-signal-intensity areas compatible with watershed infarcts).

The patterns of involvement were classified according to the involvement of cortex, white matter and deep grey matter. Analysis was performed independently and at the time of imaging blinded to the not yet determined outcome by a board certified neuroradiologist.

For each individual patient, the percentage of monitoring time with PRx ≤ 0 (intact autoregulation), PRx between 0.0 and 0.2 (borderline) and ≥ 0.2 (impaired autoregulation) was calculated as described before [3, 16]. For each of these periods, mean MAP, ICP, CPP and PRx values were determined and used to calculate the dose of each parameter according to thresholds. The respective time and percentage values were then calculated for the favorable and unfavorable outcome groups.

Additionally, in order to analyze the individual course of all above-mentioned monitoring parameters, continuous 4-h bins were created and used to calculate the respective mean values for each segment.

Statistics were analyzed using SPSS Statistics 25 (IBM, NY, USA). Continuous data were presented as mean (± SE), whereas categorical data were shown as percentages. Continuous variables were tested for equality of variances by Levene’s test. Normal distributed parametric variables like for example ICP, MAP and CPP with equal variances were compared using the unpaired or paired *t* test; otherwise, Mann–Whitney *U* test was performed. Nominal variables like for example sex, outcome group, eGOS or mechanism of cardiac arrest were tested with Fisher’s exact test. *p* values < 0.05 were regarded as significant. For multiple comparisons, Bonferroni correction was utilized via analysis of variance (ANOVA). The work has been approved by the local ethical committee; a waiver was granted for patient consent due to the retrospective analysis. Institutional board approval was granted by the Ethics Committee of University of Tuebingen (367/2016BO2).

## Results

### General

We included a total of 19 children (mean age 5.4 ± 4.5 years, range 0.2–15.3 years). Eleven children were female. No significant differences in basic patient parameters or monitoring data were found between male and female children.

Prehospital non-professional CPR was performed in 10 cases (52.6%). Prehospital professional CPR was continued in 9 of those 10 cases (47.4%). In-hospital CPR was continued in 5 cases. The other cases were either primarily in-hospital (26.3% *n* = 5) or only professional prehospital CPR was performed (*n* = 4, 21.1%). Total mean CPR duration was 31.7 ± 31.7 min (range 5–90 min). Start of invasive monitoring was immediately after admission to PICU in 15 children (78.9%) and delayed by 2–6 days in 4 (21.2%). Surgical intervention with bilateral craniectomy was performed in one child. Table 1 summarizes the basic patient characteristics and causes for cardiac arrest.

**Table 1:** Overview of patient characteristics

Sex	57.9% female (*n* = 11)			42.1% male (*n* = 8)			
Age	Mean 5.4 + / − 4.5 years (median 4.4, range 0.2–15.3 years)						
Causes of arrest	36.8% drowning (*n* = 7)	26.3% shock (*n* = 5)				26.3% hypoxia (*n* = 5)	10.5% unclear (*n* = 2)
Monitoring start after CPR	78.9% within 8 h (*n* = 15)		Each one patient at day 2, 3, 4 and 6 respectively				Mean 0.79 + / − 1.7 days
MRI	63.2% pathological (*n* = 12)			36.8% physiological (*n* = 7)			
Total monitoring time	Mean 88.0 + / − 50.1 h						
**CPR**							
Total duration	Median 15.0 min, mean 31.7 + / − 31.7 min						
Non-professional	52.6% (*n* = 10)					Median 5.0, mean 10.0 + / − 6.1 min, range 5–20 min	
Prehospital professional	47.4% (*n* = 9)					Median 14.4 min, mean 30.3 + / − 25.0 min, range 5–86 min	
In-hospital	26.3% (*n* = 5)					Median 10.3 min, mean 39.3 + / − 22.3 min, range 5–60 min	
**eGOS**							
	1	2			3–4	5–6	7–8
At discharge	42.1% (*n* = 8)	15.8% (*n* = 3)			10.5% (*n* = 2)	5.3% (*n* = 1)	26.3% (*n* = 5)
3 months	42.1% (*n* = 8)	15.8% (*n* = 3)			5.3% (*n* = 1)	5.3% (*n* = 1)	31.6% (*n* = 6)
6 months	42.1% (*n* = 8)	15.8% (*n* = 3)			5.3% (*n* = 1)	5.3% (*n* = 1)	31.6% (*n* = 6)
12 months	47.4% (*n* = 9)	10.5% (*n* = 2)			5.3% (*n* = 1)	5.3% (*n* = 1)	31.6% (*n* = 6)

### First 72 h

When analyzing the course of ICP, MAP, CPP and PRx during the first 72 h for those 15 patients who immediately received an intracranial pressure transducer, we observed that ICP in all patients with favorable outcome was continuously below 15 mmHg.

In the first 24 h, no differences in ICP, CPP and PRx were observed between outcome groups. However, ICP was higher in the unfavorable outcome group between 24 and 40 h (12.9 + / − 4.6 vs 6.4 + / − 2.5mHg, *p* = 0.021), and PRx was higher at 48–56 h (0.12 + / − 0.21 vs − 0.16 + / − 0.14, *p* = 0.014).

**Fig. 1:**
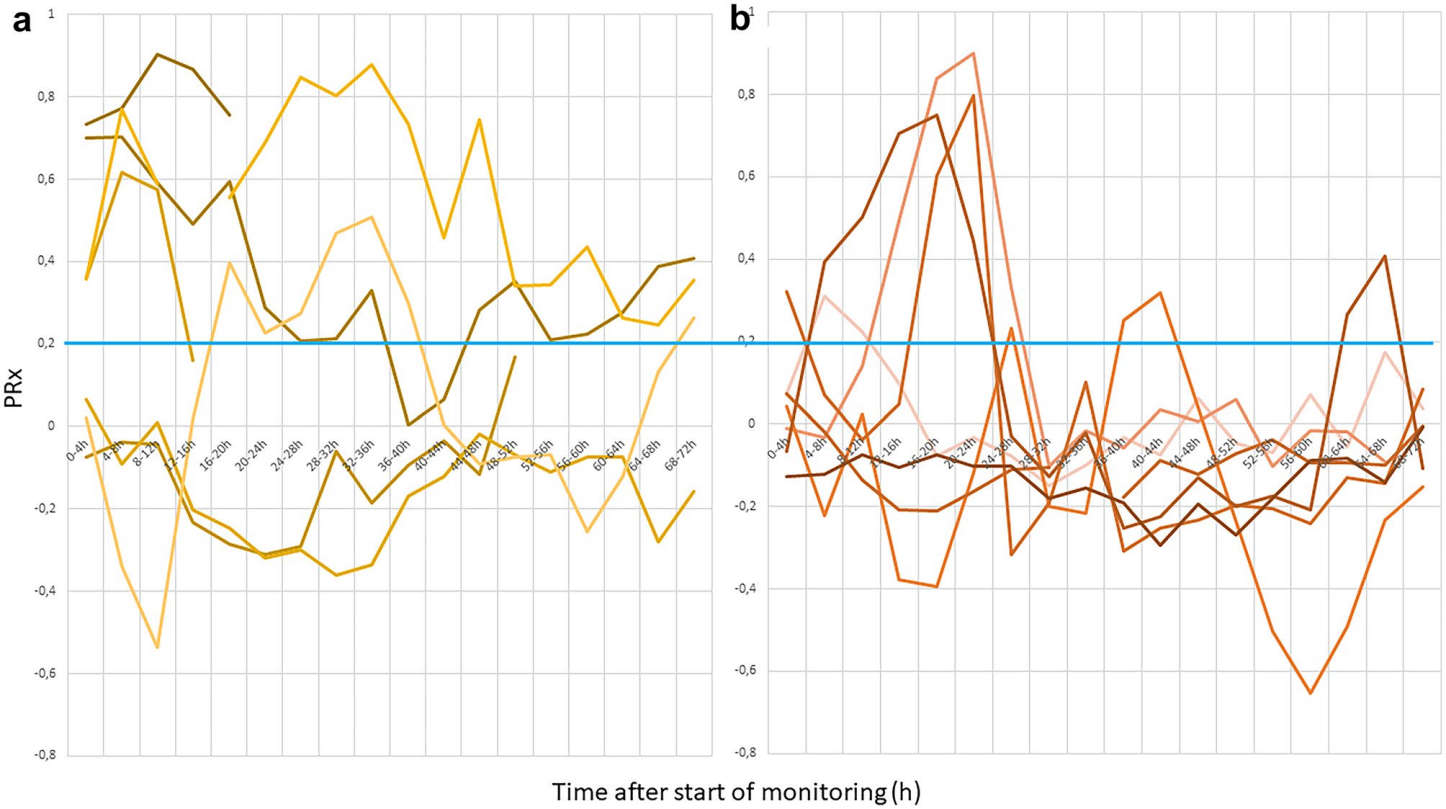
Individual course of PRx during the first 72 h of monitoring for each patient, dichotomized for **a** unfavorable (*n* = 7; one patient who deceased within 72 h was omitted from the figure), **b** favorable outcome group (*n* = 7); cutoff for PRx 0.2 indicated by horizontal blue line

**Fig. 2:**
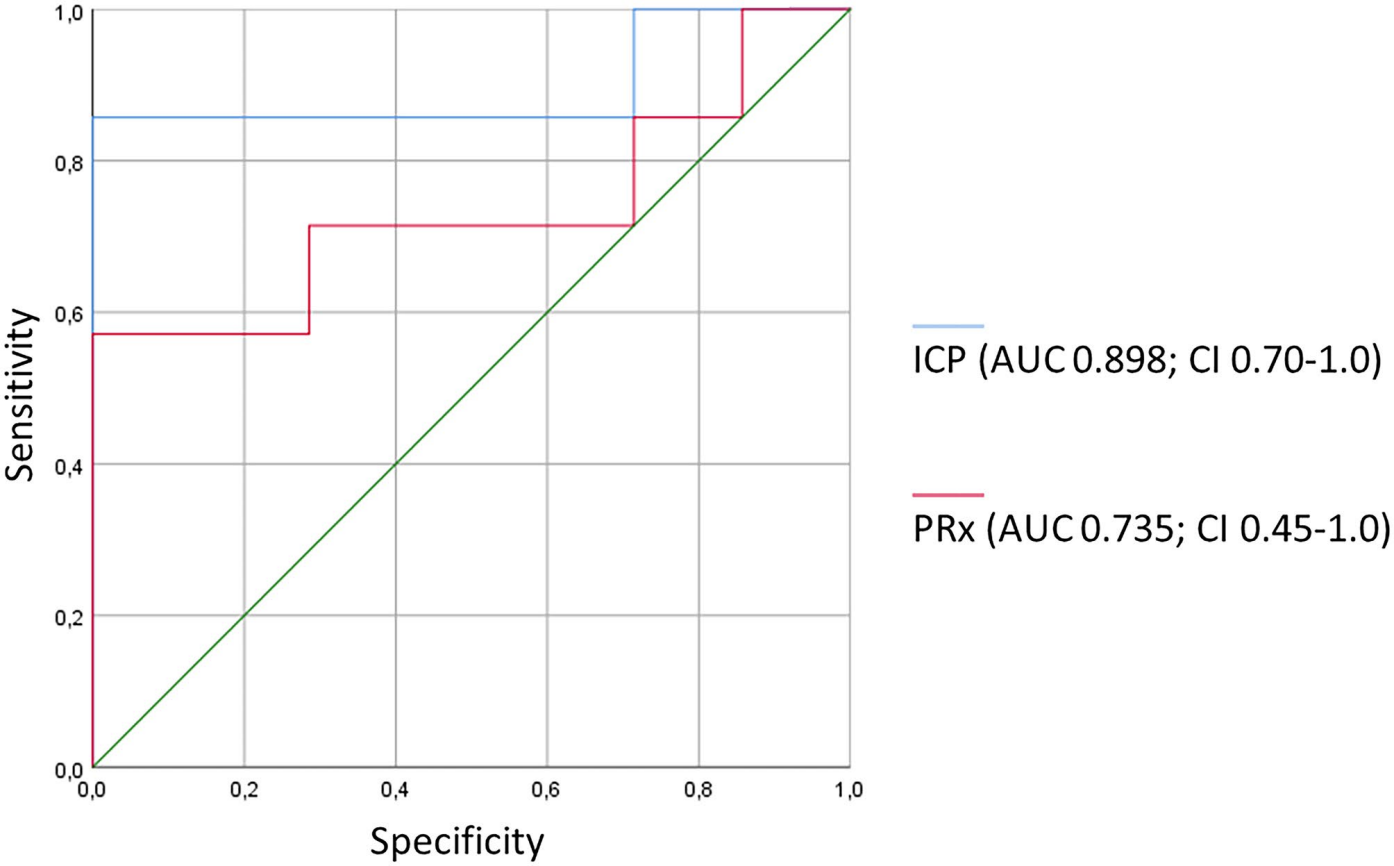
ROC analysis for ICP and PRx during first 72 h of monitoring for unfavorable outcome detection

A ROC analysis of the first 72 h of monitoring parameters revealed the following cutoff values for identification of unfavorable outcome: overall mean ICP (AUC 0.898) higher than 9.8 mmHg (85.7% sensitivity, 100% specificity) and PRx (AUC 0.735) higher than 0.2 (57.1% sensitivity, 100% specificity). See Figs. 1, 2.

### Total monitoring time

Mean monitoring time was 88.0 ± 50.1 h. Mean MAP was 74.4 ± 11.7 mmHg, mean CPP 56.2 ± 15.0 mmHg, mean ICP 18.2 ± 17.3 mmHg and mean PRx 0.15 ± 0.3. For each individual patient, three quality ranges of CAR were defined (functional PRx ≤ 0.0, borderline 0.0 < PRx < 0.2, impaired PRx ≥ 0.2). Mean total monitoring times per range were 49.4 ± 36.7 h, 4.4 ± 3.2 h and 34.2 ± 28.7 h, respectively. Relative monitoring times were 49.6 ± 25.4%, 5.1 ± 2.9% and 45.3 ± 26.7% (functional vs borderline *p* < 0.001, functional vs impaired *p* = 0.725, borderline vs impaired *p* < 0.001).

Mean MAP during these phases was 75.7 ± 10.5, 74.4 ± 12.7 and 74.1 ± 12.7 mmHg respectively (functional vs borderline p = 0.200, functional vs impaired p = 0.196, borderline vs impaired *p* = 0.694). Mean ICP was 15.0 ± 14.8, 14.9 ± 13.5 and 19.9 ± 17.8 mmHg, respectively (functional vs borderline *p* = 0.885, functional vs impaired *p* = 0.015, borderline vs impaired *p* = 0.011). The resulting mean CPP was 60.7 ± 11.7, 59.5 ± 12.6 and 54.2 ± 15.5 mmHg, respectively (functional vs borderline *p* = 0.255, functional vs impaired *p* = 0.002, borderline vs impaired *p* = 0.003). Mean PRx was − 0.31 ± 0.10, 0.14 ± 0.04 and 0.59 ± 0.18, respectively (*p* < 0.001).

### Outcome

No significant outcome differences were found between children who received professional prehospital CPR and those who did not. Eight children died during the first 2 weeks (42.1%), eGOS at discharge was 2 in 3 (15.8%), 3–4 in 2 (10.5%), 5–6 in 1 (5.3%) and 7–8 in 5 children (26.3%). At 6 months after discharge, one child improved from eGOS 4 to 7. At 12 months after discharge, another child with eGOS 2 died of secondary reasons.

**Fig. 3:**
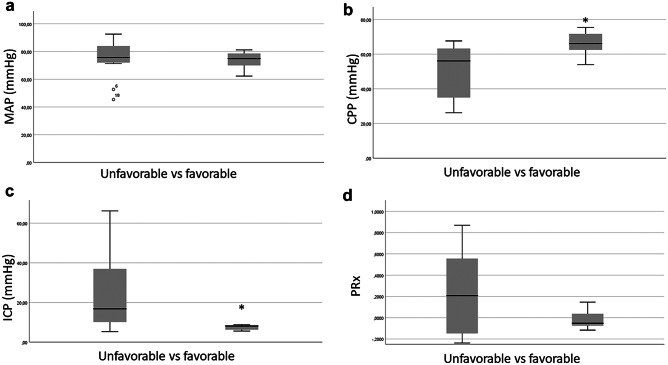
Results of mean **a** MAP, **b** CPP, **c** ICP, **d** PRx in unfavorable and favorable outcome groups *p < 0.05

The 7 patients with favorable outcome at 6 months had been compared to those who died or lived-on with unfavorable outcome: significantly shorter CPR duration (10.1 ± 10.3 vs 44.3 ± 33.4 min, *p* = 0.018), higher overall mean CPP (66.2 ± 7.6 vs 50.3 ± 15.3 mmHg, *p* = 0.021), lower ICP (7.4 ± 1.3 vs 24.5 ± 19.3 mmHg, *p* = 0.033) and tendentially lower PRx (− 0.01 ± 0.09 vs 0.24 ± 0.40, *p* = 0.118) (see Fig. 3). Table 2 summarizes the monitoring data in the unfavorable and favorable outcome group. No significant differences in core temperature, endexpiratory and arterial CO_2_ or doses of sedation were observed.

**Table 2:** Monitoring values in the unfavorable and favorable outcome group

	**Unfavorable** ***n* = 12 (63.2%)**	**Favorable** ***n* = 7 (36.8%)**	***p* value**
Total CPR time	44.3 ± 33.4 min	10.1 ± 10.3	**0.018**
Mean total CPP	50.3 ± 15.3 mmHg	66.2 ± 7.6	**0.021**
Mean total ICP	24.5 ± 19.3 mmHg	7.4 ± 1.3	**0.033**
Mean total PRx	0.24 ± 0.40	− 0.01 ± 0.09	0.118
Functional CAR (%)	41.4 ± 28.7%	63.4 ± 8.2	**0.026**
Borderline CAR (%)	3.9 ± 2.7%	7.2 ± 2.2	**0.015**
Impaired CAR (%)	54.6 ± 29.6%	29.3 ± 7.5	**0.014**

An MRI brain scan before discharge was performed in all children (> 7 days after cardiac arrest). Pathological results with significant hypoxic-ischemic brain damage were found in all children with eGOS 1–2, but not in any of the other children (*p* < 0.001). Total CPR duration was significantly longer in children with pathological MRI scan (44.3 ± 33.4 min vs 10.1 ± 10.3 min, *p* = 0.018). In addition, mean overall CPP was significantly lower (50.3 ± 15.4 mmHg vs 66.2 ± 7.6 mmHg, *p* = 0.021), and ICP was significantly higher (24.5 ± 19.3 mmHg vs. 7.4 ± 1.3, *p* = 0.033), but no differences in total PRx or MAP were observed. No significant differences concerning outcome or monitoring parameters were observed between different causes of circulatory arrest.

### "Dose” of CAR

**Fig. 4:**
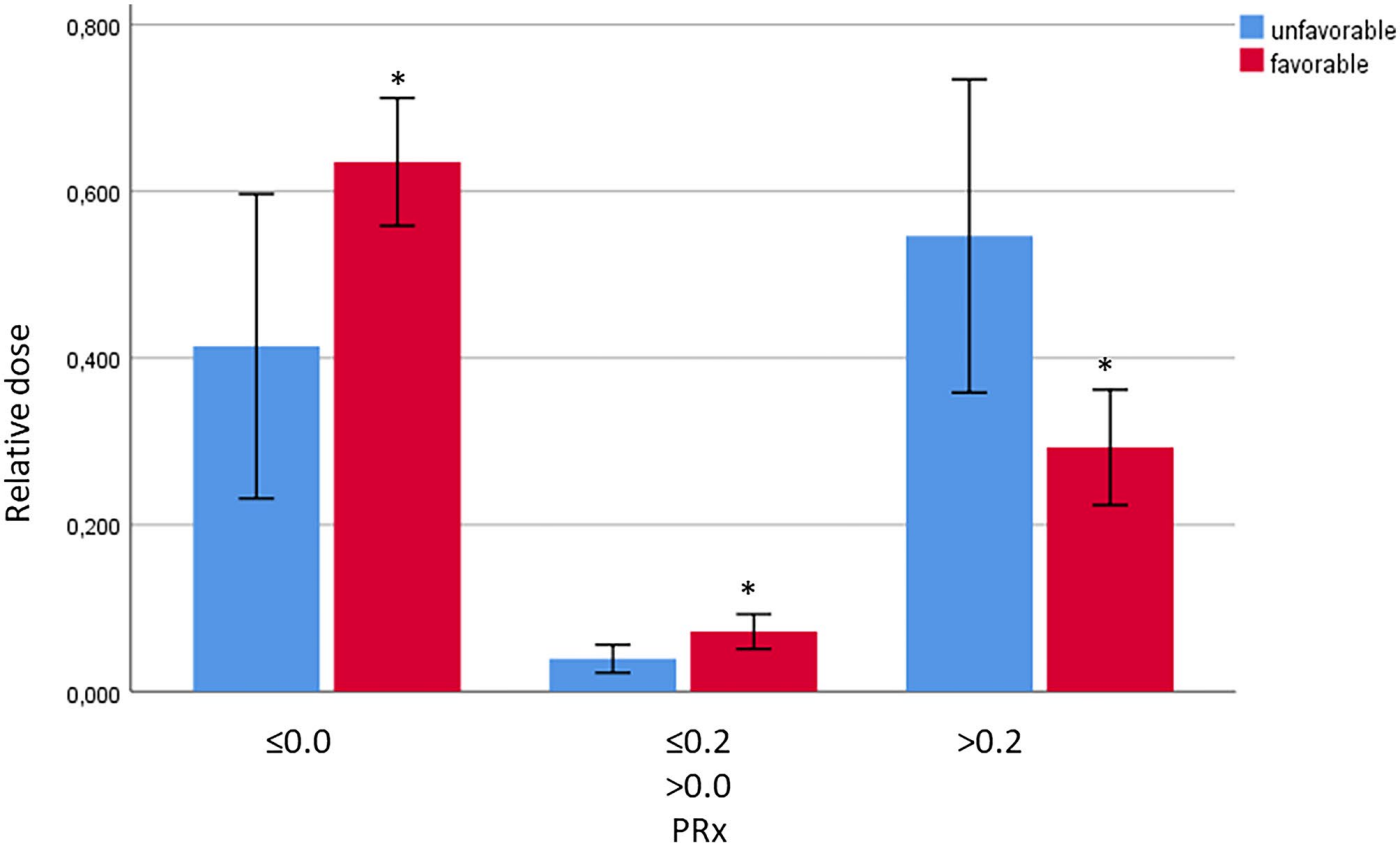
Comparison of relative monitoring times with different CAR quality, dichotomized by outcome

According to CAR quality defined by PRx (impaired/borderline/intact), relative monitoring time was compared between unfavorable and favorable outcome groups. The entire monitoring period for each patient was analyzed for the cumulative time, this patient spent with each of the PRx quality ranges. Unfavorable outcome was associated with significantly less functional CAR time (41.4 ± 28.7% vs 63.4 ± 8.2, *p* = 0.026) and borderline CAR time (3.9 ± 2.7% vs 7.2 ± 2.2, *p* = 0.015), but more impaired CAR time (54.6 ± 29.6% vs 29.3 ± 7.5, *p* = 0.014) (see Fig. 4).

## Discussion

In this pediatric cohort with a mean age of 5.4 years after cardiac arrest, we found significantly higher ICP and lower CPP in the unfavorable outcome group. Furthermore, higher PRx values were observed as a sign of impaired cerebrovascular reactivity. Whilst all patients showed episodes of intact as well as impaired CAR, the “dose” of PRx values higher than 0.2 in the first 72 h was significantly higher in children with unfavorable outcome. Unsurprisingly, longer CPR duration with higher risk of hypoxic-ischemic brain injury led to worse outcome. During the first 72 h after cardiac arrest, mean ICP higher than 10 mmHg and PRx higher than 0.2 were associated with unfavorable outcome.

There were two individual outliers to these findings: In one patient, who had—despite a persistently low PRx and ICP—an unfavorable outcome, MRI showed a limited brain ischemia mostly in the basal ganglia. The loss of this functionally important, volume-wise however minor brain area was not identified by global autoregulation monitoring with PRx. Thus, MRI remains a valuable and indispensable tool for ultimately assessing prognosis; however, definitive imaging findings may take several days to appear. Furthermore, MRI is rarely indicated and difficult to perform in the acute phase after resuscitation when children might still be fragile, and transport to MRI plus investigations under less optimal conditions than on the ICU should be avoided.

Another patient had severe neurological impairments already before cardiac arrest due to the extremely rare Peters plus syndrome. This child unfortunately died despite low ICP and PRx levels, but not because of brain ischemia.

These findings suggest that ICP monitoring after cardiac arrest is an important adjunct, since it enables, apart from reading ICP and calculating CPP, to assess the integrity/functionality of CAR. Therefore, pediatric neurosurgeons should be included in the management of these patients.

Concerning the association of persistently impaired CAR with unfavorable outcome, we were able to confirm and translate the findings of Balu et al. into the pediatric population [14]. However, other authors found an initially (in first 24 h) compromised cerebrovascular reactivity as a bad prognostic factor [17], a finding we were expecting to see, but to our surprise were not able replicate.

What we found in the unfavorable outcome group instead was an increase of ICP after 24 h (up to 40 h) preceding a more significantly deranged CAR after 48 h. The best explanation for the fact, that in our series the initial PRx (within 24 h) was not different between outcome groups, most likely is that our treatment protocol, apart from actively addressing ICP levels above 10–15 mmHg (age dependent), actively manages and guides MAP trying to achieve an optimal CPP—CPPopt—by identifying the CPP range with the best autoregulatory capacity—indicated by minimal PRx values [6]. Thus, our protocol might prevent an early dichotomization by actively addressing CPP in the first 24–40 h = first 1–2 days.

CAR is effected by vascular reactivity. The primary lethal or sublethal hypoxic-ischemic damage from the resuscitation however had its effect on the brain parenchyma, leading to a delayed cell swelling after 24 h onwards, which resulted in the ICP increase in the bad outcome group. The resulting CPP decrease as a secondary event could not be overcome and might have been leading to a deteriorating CAR. Another option is that glial and neuronal cells might have a metabolic effect on the functionality of the smooth muscle cells and thus CAR.

On the contrary, a longer time spent with a PRx below 0 (as an indicator of intact autoregulation) in the first 72 h after brain damage was associated with favorable outcome. This has already been shown for adults [18].

In summary, the inability to normalize ICP after the first 24 h, despite active attempts to decrease ICP, and to determine and reach/maintain an CPPopt, thus the inability to secondarily keep PRx below 0.2, might serve as a bad prognostic factor. Joint ICP and CAR monitoring might therefore help to prognosticate/identify those patients early, within the first 3 days, who are either likely to die or have an unfavorable outcome.

Continuous assessment of ICP and cerebrovascular reactivity via PRx, however, depends on an implanted intracranial pressure transducer. In our institution, this is part of an established routine protocol for children after cardiac arrest and resuscitation with presumed longer low flow or no flow time. The goal of this monitoring modality includes identifying elevated ICP and impaired CAR. Consequently, we use this information to optimize CPP via PRx from the very beginning, helping to avoid or at least minimize significant secondary brain damage.

Importantly, the decision for implantation of an ICP probe is not based on radiological findings (initial CT scan) but mostly clinical information. If a significant cerebral low-flow time can be assumed (due to circumstances or intermittent signs of low cerebral blood flow like dilated pupils), the decision is made irrespective of the initial CT findings, which often are not significant [19]. Certainly, if the initial CT scan already shows a severely damaged brain with loss of grey matter/white matter distinction and the child in addition shows a loss of all brain stem reflexes, this will be considered as a lethal injury and no ICP transducer will be implanted.

We cannot prove with this retrospective analysis in a small cohort, that the good outcomes in 7 of 19 patients were attributable to the CPP optimization protocol. However, emerging prospective data in cardiac arrest suggests so [20], which is in accordance with findings from TBI.

The limitations of this study include the small sample size, a heterogenous cohort and furthermore a broad range of reasons for cardiac arrest as well as CPR duration. Additionally, in 4 patients of our cohort, monitoring was delayed by more than 24 h. Subsequent data analysis may have been influenced by this fact; therefore, a separate analysis of patients with immediate implantation of ICP probe was performed for the first 72 h, and therefore the main results have only been generated from 15 patients.

Despite the multitude of CAR data, autoregulation still cannot be equated with cerebral blood flow regulation. Other mechanisms such as neurovascular coupling, carbon dioxide-induced vascular reactivity, myocardial function, autonomic nervous system control and the role of the neurovascular unit have to be considered [21]. Targeted neuroprotective agents are not yet available [22] though promising experimental data is available [23, 24]. Factors such a ZNF580 [25] or erythropoietin have shown promising roles in neuroprotection [26]. Emerging data on the role of agents such as nerve growth factor with reports of experimental use have been reported [27].

Furthermore, hypoxia-inducible factor signaling pathways are involved in genetic transcription during hypoxic-ischemic preconditioning. For example, these effects include vasodilatation and vascoconstriction, neoangiogenesis and anti-apoptosis [28, 29]. Other confounders mediated by interventions optimizing CPP may contribute to good outcome. Diffusion limitation of oxygen delivery at the level of the neurovascular unit exists, indicating that macrovascular oxygen delivery to the brain tissue alone is probably not the sole determinant of favorable outcome [30]. On the other hand, recent literature suggests that variations in cerebral perfusion are associated with changes in cerebral lactate/pyruvate ratio [31].

Larger cohort studies, ideally prospectively randomized multicenter studies, need to investigate the routine use of ICP and thus PRx monitoring and CPP optimization in post-cardiac arrest/post resuscitation children.

## Conclusion

Significant doses of impaired CAR between 24 and 72 h after cardiac arrest are associated with unfavorable outcome. Bad prognostic signs are the inability to restore or maintain functional autoregulation despite active attempts to do so as well as an increase of ICP above 15 mmHg. However, limited ischemia might not be detected by ICP-based autoregulation monitoring and can still result in unfavorable outcome despite good global autoregulation.

## Supplementary information

Below is the link to the electronic supplementary material.

Supplementary file1 (TIF 148 KB)

Supplementary file2 (DOCX 20 KB)

Supplementary file3 (DOCX 17 KB)
