# Supplementary figures and images for: Monitoring of cerebrovascular pressure reactivity in children may predict neurologic outcome after hypoxic-ischemic brain injury
Source: Childs Nerv Syst. 2022 Jun 9;38(9):1717–26. doi: 10.1007/s00381-022-05579-4 (PMC9463308; doi:10.1007/s00381-022-05579-4)

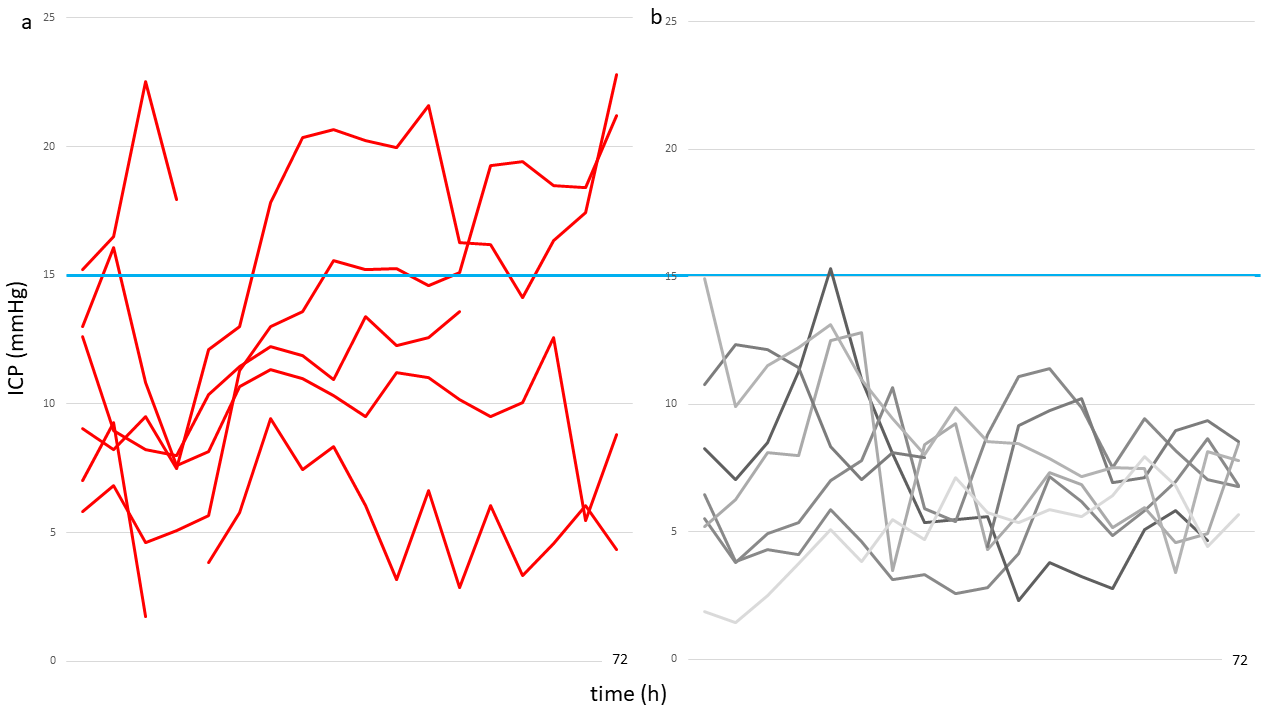

Supplement: Supplementary file 1 — Supplementary file1 (TIF 148 KB) [file 381_2022_5579_MOESM1_ESM.tif]
