# Supplementary material for: Monitoring of cerebrovascular pressure reactivity in children may predict neurologic outcome after hypoxic-ischemic brain injury
Source: Childs Nerv Syst. 2022 Jun 9;38(9):1717–26. doi: 10.1007/s00381-022-05579-4 (PMC9463308; doi:10.1007/s00381-022-05579-4)
Supplement: Supplementary file 2 — Supplementary file2 (DOCX 20 KB) [file 381_2022_5579_MOESM2_ESM.docx]

Supplemental table1:

Overview of patient characteristics and causes for cardiac arrest.

| no. | age (years) | Mechanism | Duration of CPR (min) | Monitoring duration (h) | eGOS0 | eGOS3 | eGOS6 | eGOS12 |
| --- | --- | --- | --- | --- | --- | --- | --- | --- |
| #1 | 15.3 | hypoxia | 40 | 19.1 | 1 | 1 | 1 | 1 |
| #2 | 4.8 | hypoxia following air embolism | 5 | 162.5 | 1 | 1 | 1 | 1 |
| #3 | 14.3 | drowning following epileptic attack | 10 | 92.4 | 7 | 7 | 7 | 7 |
| #4 | 2.25 | unclear | 15 | 47.4 | 1 | 1 | 1 | 1 |
| #5 | 2.4 | shock (intestinal volvulus) | 40 | 69 | 1 | 1 | 1 | 1 |
| #6 | 4.4 | propofol infusion syndrome, cardiogenic shock | 60 | 11.9 | 1 | 1 | 1 | 1 |
| #7 | 5.2 | drowning | 18 | 79.7 | 2 | 2 | 2 | 2 |
| #8 | 2.75 | hypoxia after seizure | 0 | 64 | 4 | 7 | 7 | 7 |
| #9 | 0.45 | hypoxia due to subglottical stenosis | 10 | 158 | 1 | 1 | 1 | 1 |
| #10 | 1.9 | drowning | 30 | 179.4 | 7 | 8 | 8 | 8 |
| #11 | 8.7 | aspiration, hypoxia | 8 | 35.6 | 1 | 1 | 1 | 1 |
| #12 | 1.5 | drowning | 90 | 101 | 1 | 1 | 1 | 1 |
| #13 | 5.3 | drowning | 6 | 112.2 | 8 | 8 | 8 | 8 |
| #14 | 10.3 | hemorrhagic shock | 0 | 112.4 | 8 | 8 | 8 | 8 |
| #15 | 7.3 | hemorrhagic shock | 80 | 112.8 | 3 | 3 | 3 | 3 |
| #16 | 1.2 | drowning | 10 | 102 | 7 | 7 | 8 | 8 |
| #17 | 0.2 | septic shock | 86 | 104.1 | 2 | 2 | 2 | 1 |
| #18 | 4.25 | unclear | 80 | 0.5 | 2 | 2 | 2 | 2 |
| #19 | 11 | drowning | 15 | 108.4 | 7 | 7 | 7 | 7 |

Supplemental table2

Comparison of monitoring episodes defined by PRx

|  | total | functional CAR | borderline CAR | impaired CAR |
| --- | --- | --- | --- | --- |
| relative monitoring time | 100% | 49.6% | 5.1% | 45.3% |
| mean PRx | 0.15±0.3 | -0.31±0.10 | 0.14±0.04 | 0.59±0.18 |
| mean MAP | 74.4±11.7 | 75.7±10.5 | 74.4±12.7 | 74.1±12.7 |
| mean CPP | 56.2±15.0 | 60.7±11.7 | 59.5±12.6 | 54.2±15.5 |
| mean ICP | 18.2±17.3 | 15.0±14.8 | 14.9±13.5 | 19.9±17.8 |
