# Supplementary material for: Monitoring of cerebrovascular pressure reactivity in children may predict neurologic outcome after hypoxic-ischemic brain injury
Source: Childs Nerv Syst. 2022 Jun 9;38(9):1717–26. doi: 10.1007/s00381-022-05579-4 (PMC9463308; doi:10.1007/s00381-022-05579-4)
Supplement: Supplementary file 3 — Supplementary file3 (DOCX 17 KB) [file 381_2022_5579_MOESM3_ESM.docx]

**Pediatric Post-Resuscitation Care Protocol (PPRCP)**

This protocol is valid for all pediatric patients outside the neonatal period with an initial GCS < 8

**Monitoring:**

- SpO2 by pulse oximetry
- quantitative end tidal CO2 or capnography
- intra-arterial arterial blood pressure
- ECG
- continuous core temperature
- urine output
- blood glucose
- arterial and central venous blood gas (pH, paCO2, paO2)
- arterial serum lactate
- electrolytes
- creatinine
- complete blood count
- coagulation profile
- ICP, CPP and CAR-Monitoring
- EEG
- brain CT at admission

**Targets:**

- Optimize oxygenation and ventilation: the lowest fraction of inspired oxygen (FiO2) should be used to maintain an oxygen saturation of 94% - 99% (paO2 80-100 mmHg; paCO2 35-38 mmHg)
- Optimize cardiac output by ensuring adequate preload and contractility according to the hemodynamic protocol
- Avoid hypotension: neonates MAP > 40 mmHg, infants MAP > 45 – 50 mmHg, toddler MAP > 50 – 55 mmHg, schoolchild MAP > 60 mmHg; adult MAP > 70 mmHg
- Treat arrhythmias, maintain heart frequent adequate for age
- Maintain adequate hemoglobin concentrations (Hb 9,5 - 10 g/dl)
- Avoid hyperthermia by active temperature management with the primary aim of strict normothermia (36-36.5 °C) for 72 hours after cardiopulmonary arrest
- Avoid hypoglycemia (≤45 mg/dL in the newborn and ≤60 mg/dL in the child)
- Avoid electrolyte imbalance
- Treat seizures
- Maintain ICP < 15 mmHg in children and < 20 mmHg in adults: 3% hypertonic saline (NaCl 3%) 2-5 ml/kg over 10-20 min; avoid persistent serum sodium > 165 mmol/l
- Maintain CPPopt via CAR-Monitoring, if no CPPopt is available maintain CPP > 40-45 mmHg in infants, CPP > 45-48 mmHg in toddler 1-2 years, CPP > 48 mmHg in children 2-6 years, CPP > 54 mmHg in children 7-10 years, CPP > 58 mmHg in teenager 11-15 years and CPP > 70 mmHg in adults -re-evaluation every 4 hours

Supplemental figure legend

Course of ICP during the first 72 hours of monitoring, unfavorable vs favorable outcome group
